# Heterogeneous distribution of dye-labelled biomineralizaiton proteins in calcite crystals

**DOI:** 10.1038/srep18338

**Published:** 2015-12-17

**Authors:** Chuang Liu, Liping Xie, Rongqing Zhang

**Affiliations:** 1 Institute of Marine Biotechnology, Collaborative Innovation Center of Deep Sea Biology, School of Life Sciences, Tsinghua University, Beijing 100084 China; 2 Tsinghua-Peking Joint Center for Life Sciences, School of Life Sciences, Tsinghua University, Beijing 100084 China

## Abstract

Biominerals are highly ordered crystals mediated by organic matters especially proteins in organisms. However, how specific proteins are distributed inside biominerals are not well understood. In the present study, we use fluorescein isothiocyanate (FITC) to label extracted proteins from the shells of bivalve *Pinctada fucata.* By confocal laser scanning microscopy (CLSM), we observe a heterogeneous distribution of dye-labelled proteins inside synthetic calcite at the microscale. Proteins from the prismatic calcite layers accumulate at the edge of crystals while proteins from the nacreous aragonite layers accumulate at the center of crystals. Raman and X-ray powder diffraction show that both the proteins cannot alter the crystal phase. Scanning electron microscope demonstrates both proteins are able to affect the crystal morphology. This study may provide a direct approach for the visualization of protein distributions in crystals by small-molecule dye-labelled proteins as the additives in the crystallization process and improve our understanding of intracrystalline proteins distribution in biogenic calcites.

Organisms can control the crystallization and self-assembly of biominerals at a range of scales spatially and temporally. Biominerals usually have superior materials properties such as high crack resistance and are used for protection, navigation, ion storage and light scattering^1^,^2^. Therefore, to understand the regulators of biomineralization can provide information for synthesis of bioinspired materials with remarkable properties. Organic matters are known to play critical roles in the nucleation, crystallization and growth of biominerals^3^. Among them, proteins have the major impact on the formation of biominerals. Proteins extracted from biominerals influence the crystallization process, from the precipitation rate to the polymorph and morphology of CaCO_3_ crystallite^4^,^5^,^6^, which is the most abundant biogenic biominerals.

Although identification of biomineralization proteins is a prerequisite to better understand biomineralization, their spatial location and function are equally important. Deciphering the spatial location of these proteins is challenging because of their small size and diversity. Annular dark-field scanning transmission electron microscopy (ADF-STEM) coupled with three-dimensional (3D) reconstruction have been used for nanoscale visualization of biomacromolecules within the calcite from *Atrina rigida* shells^7^ and calcite single crystals grown in agarose hydrogels^8^. The organic matters are found to present as network or aggregates inside the calcite^8^. Electron tomography has also been used to show organics-containing voids in the biominerals^9^,^10^. X-ray absorption near-edge structure (XANES) spectroscopy is used to characterize the organic contents along the axis of prisms, indicating the presence of concentrated organic regions^11^. XANES has also been used to show asprich peptides can be occluded in calcite and permanently disorder biomineral crystals at short- and long-range distances^12^. Pulsed-laser atom-probe tomography (APT) has been explored to construct 3D chemical maps of the organic fibers in nanocrystalline magnetite mineral in the tooth of the chiton *Chaetopleura apiculata*^13^. These methods enable us to visualize 3D structures of organics occluded in biominerals, but have limitations on showing how one type of biomacromolecule is distributed. X-ray powder diffraction (XRD) is a powerful tool to show that incorporation of organic matters into biominerals can cause anisotropic lattice distortions along the c-axis compared with their nonbiogenic counterparts^14^,^15^. Using this method, Boaz Pokroy *et al.* screened the incorporation of 20 amino acids into calcite and they found that amino acid charge, size, rigidity and the relative pK_a_ of the carboxyl and amino functional groups were important factors for the interaction with the mineral phase^16^.

As a complementary method, Weber *et al.* recently use recombinant green fluorescent protein (GFP)-fusion proteins to study the effects of perlucin- a lectin protein from the nacre of mollusk *Haliotis lavigata*- on the crystallization of CaCO_3_ and visualize them by confocal laser scanning microscopy (CLSM)^17^,^18^. By XRD, they find that GFP-perlucin can be incorporated into the calcite and induce concentration dependent anisotropic lattice distortions along the host’s c-axis^18^. However, GFP is a relatively large protein with a molecular weight (MW) of ~26.9 kDa compared to ~17.9 kDa perlucin and GFP has an isoelectric point of 5.67^17^. Acidic proteins are known to affect the crystallization process^19^,^20^. Last but not the least, some researchers use the immunolocalization approach, in which antibodies against biomineralization proteins are used to localize the specific proteins in the shells of *Pinna nobilis*^21^ and to observe skeletal matrix proteins in the mineral of the coral *Stylophora pistillata*^22^. This method can indirectly observe the spatial relationship between the organic and mineral phases, but it cannot reflect how proteins are distributed within the mineral.

Another possible approach to visualize proteins in crystals is to label macromolecules with small fluorescent molecules^23^,^24^. Fluorescein isothiocyanate (FITC) is a derivative of fluorescein with a MW of 389.38 Da and has excitation and emission spectrum peak wavelengths of approximately 495 nm and 519 nm. In order to probe the distribution mode of biomineralization proteins inside crystals, we use FITC to label proteins extracted from the shells of bivalve *Pinctada fucata*. The shell of the pearl oyster, *P.fucata*, which is widely used to produce saltwater pearls in China and East Asia, consists of two different forms of calcium carbonate, i.e. calcite in the outer prismatic layer and aragonite in the inner nacreous layer^25^. They have been extensively used as a biomineralization model to explore relationship between macromolecules and calcium carbonate crystallization^26^,^27^. By CLSM in 2D mode and Z-stack imaging, we are able to determine the distribution of FITC-labelled proteins inside the artificial calcite according to their fluorescence intensity. The results show that proteins from different parts of shells have different distribution patterns inside the synthetic calcite, which may reflect their different roles in CaCO_3_ crystal growth. The results also raise a possibility that different proteins controlling crystal growth in natural biominerals have different distributions and locations.

## Methods

### Shell preparation and protein extraction

All methods were carried out in accordance with the approved guidelines. All experimental protocols were approved by the Animal Experimental Ethics Committee of Tsinghua University, Beijing, China.

Pearl oysters, *Pinctada fucata* (with shells of 5.5–6.5 cm in length, 30–40 g of wet weight, and approximately two years of age), were obtained from Zhanjiang Pearl Farm (Guangxi Province, China). The cleaned shells of *P. fucata* were immersed in a 5% NaOH solution for 24 h followed by rinsing with distilled water to avoid possible contamination from soft tissues that may have adhered to the inner surface of shells. The two layers of the shells, the outer calcitic prismatic layer and the inner aragonitic nacreous layer, were separated mechanically by abrasion after air-drying. The fragments were pulverized (30 g) and then decalcified with 0.8 M EDTA at 4 °C for 60 h with continuous stirring. For the extraction of the EDTA soluble matrix (ESM-P/N, P and N are prismatic and nacreous layers respectively), the supernatant was collected by centrifugation at 13,000 rpm at 4 °C for 30 min and then desalted by ultrafiltration (3 K, 4000 rpm, 1–2 h). The obtained samples were applied on 12% SDS-polyacrylamide gels and stained with Coomassie Brilliant Blue R250. The concentration of proteins was determined by Nanodrop 2000.

### Preparation of FITC-labelled proteins

Proteins (1 mL, 250 μg·mL^−1^) were mixed with 75 μL FITC (dissolved in DMSO, 1 mg·mL^−1^) and then incubated for 1 h, followed by dialysis against DI water for 72 h with exchange of water every 24 h. The entire process was conducted in the dark^28^. The resultant proteins are designated as FITC-ESM-P/N.

### Crystallization of CaCO_3_ using FITC-proteins as the additives

Calcium carbonate was deposited by the slow diffusion of ~2 g (NH_4_)_2_CO_3_ vapor into six cell-culture dishes in a closed desiccator for ~14 h at room temperature. Each well contained 180 μL of 20 mM CaCl_2_ and 20 μL proteins with a concentration of ~25 μg·mL^−1^ on a silica cover glass. The proteins are ESM-P/N or FITC-ESM-P/N. For the control, the proteins were replaced with DI water or FITC. The samples for further test were rinsed twice with DI water and air-dried.

### Characterization

The morphologies of clean samples were observed by a scanning electron microscope (SEM, FEI Quanta 200, 15 kV) after sputter-coating with a thin layer of gold nanoparticles. The Raman spectra were recorded at an excitation wavelength of 514 nm. The spectra were scanned three times for 20 s in the range 100–1400 cm^−1^ using a LabRAM HR Evolution spectrometer (HORIBA Jobin Yvon). The CLSM images were obtained using a LSM 710 META confocal Microscope (Zeiss), and the excitation wavelength for FITC-labeled samples was set at 488 nm. The X-ray diffraction (XRD) patterns were obtained using a Bruker D8 Advance X-ray diffractometer (Cu Kα, λ = 0.154 nm, 40 kV, 200 mA). The scattered radiation was detected in the angular range 20−80° (2θ) with a scan rate of 1.2°·min^−1^. Unit cell parameters were calculated by Jade 5. The circular dichroism (CD) measurements were carried out using a Chirascan plus ACD spectropolarimeter (Applied Photophysics) using 5-mm path length cuvettes.

## Results

**Figure 1:**
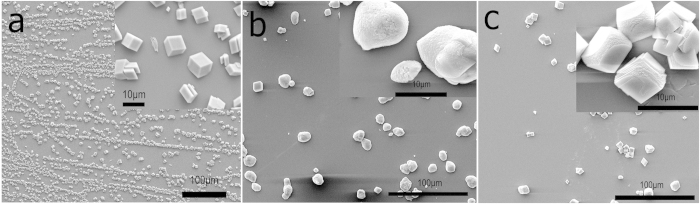
Scanning electron microscope (SEM) images of CaCO_3_ crystals obtained without the addition of any proteins (**a**) and in the presence of ~2.5 μg·mL^−1^ ESM-P (**b**) and ESM-N (**c**). Insets are their magnified images. ESM-P: EDTA soluble matrix from the prismatic layers of *Pinctada fucata*; ESM-N EDTA soluble matrix from the nacreous layers of *Pinctada fucata*.

The extracted proteins were divided into two groups according to their origin from the prismatic calcite or nacreous aragonite layer: ESM-P and ESM-N (ESM: EDTA-soluble matrix; P: prismatic layer; N: nacreous layer). Before determining the location and distribution of these proteins in synthesized CaCO_3_, their effects on the crystal growth and morphology were investigated. Compared with the rhombohedral crystals of the control sample (Fig. 1a), ESM-P significantly affected the morphology of CaCO_3_, forming round crystals with diameters of 5–10 μm (Fig. 1b). Moreover, the surfaces of the crystals were rougher than the control. When ESM-N was used as the additive, truncated patterns were obtained at the edge of the crystals (Fig. 1c).

**Figure 2:**
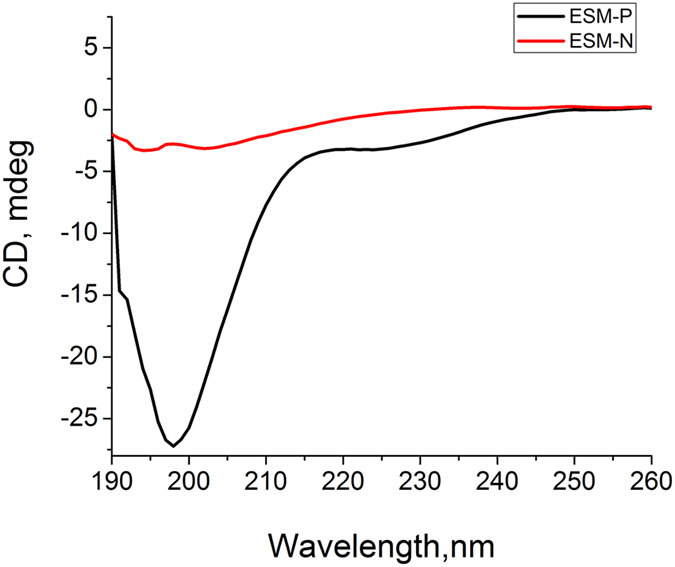
Circular dichroism (CD) spectra of ESM-P and ESM-N.

CaCO_3_ with different morphology formed in the presence of protein fractions from the shell may be caused by different protein functions. Protein conformation are known to be closely related to their functions. Therefore, the average secondary structures of proteins were determined by circular dichroism (CD) measurements (Fig. 2). ESM-P showed a typical disordered structure with a minimum negative peak at 198 nm and a value close to zero at 222 nm. ESM-N had minimum peak below 200 nm, indicating a disordered structure. It is noteworthy that ESM-P has [θ]_198_ of −27 mdeg while ESM-N has [θ]_198_ of −2.5 mdeg, indicating ESM-P has higher degree of disorder.

To monitor the initial stage of crystal formation, a pH-drop assay was performed (Figure S1). The formation of CaCO_3_ by CaCl_2_ and NaHCO_3_ generated H^+^, thus decreasing the pH of the solutions. The final concentration of proteins was ~2.5 μg·mL^−1^. After the addition of CaCl_2_, the pH of NaHCO_3_ solution rapidly decreased from 8.7 to 7.4. In the next 10 min, the pH of the control solution finally remained at 7.0. In comparison, ESM-N/P inhibited the precipitation of CaCO_3_, as evident from their final pH at 7.1 and 7.7, indicating ESM-P has more inhibitory effect over CaCO_3_ growth at the initial stage of crystal formation.

**Figure 3:**
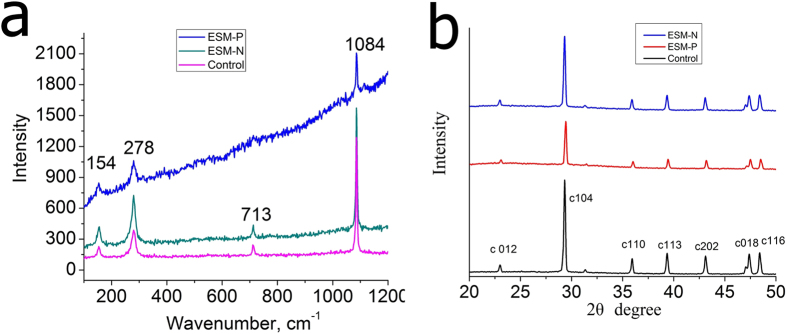
(**a**) Raman spectra of the control sample, and CaCO_3_ in the presence of ~2.5 μg·mL^−1^ ESM-P and ESM-N proteins. (**b**) X-ray diffraction (XRD) patterns of the control sample, and CaCO_3_ in the presence of ~2.5 μg·mL^−1^ ESM-P and ESM-N proteins with 2θ degree from 20 to 50.

The Raman spectra show that ESM-P and ESM-N proteins at this concentration yielded calcite, as evident from their peaks at 154, 278, 713, and 1084 cm^−1^ (Fig. 3a). The crystallization behaviour of CaCO_3_ using different protein extracts as the additives was analyzed by XRD. As shown in Fig. 3b, in agreement with Raman data, all the precipitated CaCO_3_ exhibited characteristic peaks of calcite (JCPDS No. 05-0586). Based on the full width at half maximum (FWHM) of the (104) diffraction peak, the crystal size of calcites of the control, ESM-P, and ESM-N samples were calculated to be approximately 90.3, 70.2, and 73.8 nm by the Scherrer equation. Thus, all the crystal sizes decreased, and the crystal size of the ESM-P sample decreased the most. Previous reports demonstrated that the incorporation of proteins into the crystalline lattice of calcite could reduce the grain size^18^.

**Figure 4:**
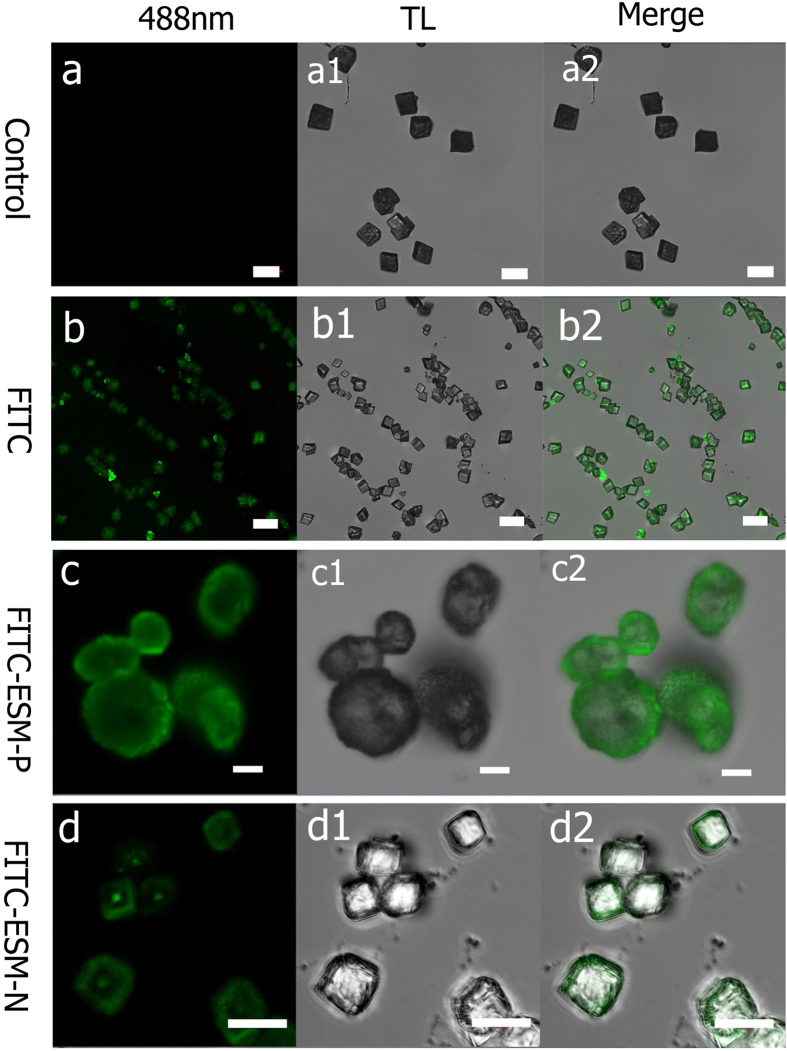
Confocal laser scanning micrographs of CaCO_3_ precipitates (**a, a1, a2**) the control CaCO_3_ formed without the addition of proteins. (**b, b1, b2**) the CaCO_3_ formed in the presence of fluorescein isothiocyanate (FITC). (**c, c1, c2**) the CaCO_3_ formed in the presence of FITC-labelled ESM-P proteins. (**d, d1, d2**) the CaCO_3_ formed in the presence of FITC-labelled ESM-N proteins. (**a–c**) are images taken at 488 nm lasers excitation (intensity, 18%); a1, b1 and c1 are bright-field images; a2, b2 and c2 are the merged images. Scar bars, a-a2 and b-b2, 20 μm; c-c2 and d-d2, 5 μm.

**Figure 5:**
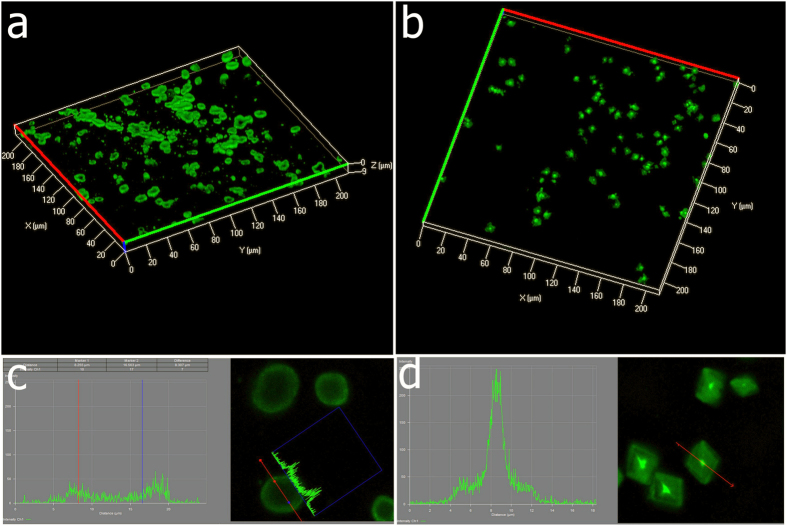
Z-stack images of CaCO_3_ formed in the presence of FITC-labelled ESM-P (**a**) and ESM-N proteins (**b**) (scanning depth 9 μm, interval 0.75 μm, area 200 × 200 μm^2^). Fluorescence signals intensity across the lines in the crystals in the presence of FITC-labelled ESM-P (**c**) and ESM-N proteins (**d**). (The analysis were performed using ZEN 2012, Zeiss).

Confocal laser scanning microscopy (CLSM) is a technique for obtaining high-resolution optical images with depth selectivity, which allows imaging at a controlled and highly limited depth of focus^29^. CLSM is widely used in numerous biological science disciplines, from cell biology and genetics to microbiology and developmental biology by labelling biological objects with fluorescent markers^29^. Therefore, CLSM can be a powerful tool to visualize interaction between organic matters with inorganic materials such as crystals^23^,^30^. Figure 4 shows the confocal laser scanning micrograph of calcium carbonate precipitated with dye-labelled proteins as the additives. FITC was used because of its availability and small molecular weights compared to GFP. FITC is negatively charged and has a deprotonated carboxyl group on the phenyl ring at pH 8.0 (the pH of reaction solution). In the control group in which no additives were added, no fluorescence was observed at 488 nm with intensity of 18% (Fig. 4a-a2). Under the same condition, when FITC was added in the CaCl_2_, homogenous fluorescence was observed inside the calcite (Fig. 4b-b2) and the morphology was identical to the control group, indicating FITC has no preference binding to specific faces of calcite. Interestingly, when FITC-ESM-P protein was added, the rounded particles were formed and heterogeneous fluorescence was observed. Specifically, the fluorescence was more concentrated at the edge of particles (0.6–1.7 μm in depth below the surfaces of particles) (Fig. 4c-c2). When FITC-ESM-N protein was added, rhombohedra crystals with blur surfaces under optical microscope were formed and heterogeneous fluorescence was found. The fluorescence was concentrated at the center (with an area of 1–4 μm^2^) of crystals (Fig. 4d–d2). To exclude the possible misinterpretation caused by the different orientation of CaCO_3_ on the glass cover, we performed z-stack imaging in a region of 200 × 200 μm^2^ with 9 μm height (interval 0.75 μm). Calcites in the ESM-P group showed a hole in the center of globular particles (Fig. 5a); calcites in the ESM-N group showed strong fluorescence at the center of crystals (Fig. 5b), which is in agreement with Fig. 3d. The z-stacking images also confirmed that the extracted proteins were not just present on the crystal surfaces but actually occluded inside the crystals. Fig. 5c shows that in the ESM-P group, fluorescence intensity at the edge of particles is 2–5 times than the other parts. By contrast, in the ESM-N group, fluorescence intensity at the center of particles is 5–10 times than the other parts (Fig. 5d).

## Discussion

Crystal formation in the presence of protein extracts have been studied before. An interesting experiment found that nacreous organic extracts of *Pinctada margaritifera* at concentration above 25 μg·mL^−1^ induced hierarchical CaCO_3_ with nanoparticles and nanoplates^4^. However, in our work, no hierarchical structure at the microscale was observed, which was likely due to the concentration of proteins used was low (~2.5 μg·mL^−1^). In addition, some investigations showed that proteins extracted from nacreous layers led to formation of aragonite *in vitro*, which could be caused by the reaction conditions such as the addition of β-chitin or silk fibroin^3^. In the present study, no other additives were added in the reaction solution except the extracted proteins. Another possibility is related to biological species. Recently studies suggested that biomineralization process in the coral was species specific. In an *in vitro* mineralization system without magnesium ions, intraskeletal organic matrix (OM) extracted from *Balanophyllia europaea* favoured the precipitation of aragonite while OM from other corals favoured calcite^31^. Therefore, it is not surprising that only calcite was formed in the presence of ESM-P or ESM-N of *P.fucata*.

Intracrystalline molecules in biogenic crystals and synthetic crystals are investigated by various approaches. By using annular dark-field scanning (ADF)-STEM, Hanying Li *et al.* have observed that in the synthetic single crystals, the incorporated agarose aggregates were distributed as a 3D network of nanofibers as opposed to isolated molecules or individual fibers. These nanofibers became cavities after heat pyrolysis^8^. A similar approach was employed by Saeed Younis *et al.* to investigate distribution of organic inclusions within individual aragonitic lamellae of *Perna canaliculus*. They found that intracrystalline organic inclusions ranging in sizes between 2 to 25 nm were distributed within the entire volume of the lamellae and 50 nm zones adjacent to the organic sheets between lamellae were almost free of inclusions^9^. The distribution mode may facilitate crack energy dissipation within individual lamellae. Combining transmission electron microscopy, electron tomography, energy-dispersive X-ray analysis and electron energy-loss spectroscopy, Katharina Gries *et al.* found 2.5–38.4 nm carbon-rich voids in the aragonite platelets of *Haliotis laevigata* nacre^10^. By atomic force microscopy (AFM), organic matrix in the tablet of *Pinctada maxima* nacre was visualized as continuous organic framework encapsulating nanograins^32^. The above methods reflect the distribution of overall organic matter in the biominerals or synthetic calcite. By using recombinant perlucin-GFP proteins as additives, one can use CLSM to visualize their distribution inside calcite. The perlucin-GFP proteins were found to induce concentration-dependent anisotropic lattice distortions along the host’s c-axis^18^ and were accumulated preferably at the interfaces between the lamellae^17^.

Using other molecules instead of proteins as occlusion matter can provide unique models for understanding occlusion of molecules in biominerals formation. A unique model was demonstrated by Fiona C.Meldrum *et al.* They incorporated 20 nm carboxylic acid-functionalized diblock copolymer micelles which acted as pseudo-proteins within single crystals of calcite. The micelles were randomly distributed inside crystals, broadening {104} diffraction peaks, and hardening the crystals^33^.

Compared with their results, we found that extracted proteins from prismatic and nacreous layers shells of *P.fucata* are heterogeneously distributed inside synthetic calcites. Rather than random and uniform distribution, the proteins have different distribution patterns: the proteins from prismatic layer are accumulated at the edge of crystals while the proteins from the nacreous layer are accumulated at the center. Interestingly, using TEM and electron energy-loss spectroscopy, Taiga Okumura *et al.* have shown that incorporated intracrystalline organic molecules (IOMs) of *P.fucata* are distributed inhomogeneously to form small-angle grain boundaries inside the calcitic prisms. In addition, IOMs extracted from the prisms are inhomogeneously incorporated into synthetic calcites^34^. By contrast, IOMs of another species *Atrina pectinata* are distributed almost homogeneously in the biominerals and in the synthetic calcite^34^. These results indicate that protein distributions in biominerals are species-specific. Especially, inhomogeneous proteins distribution in biominerals or synthetic calcite may occur from the nanoscale as observed by Taiga Okumura to the microscale as observed by us.

CD spectra show that the two protein groups are both predominantly composed of disordered structures. Through bioinformatics analysis, J. Evans found that almost all the biomineralization associated protein sequences contained at least one region of intrinsic disorder with the highest percentages found in the framework and pearl-associated proteins relative to the intracrystalline proteins^35^. Disordered proteins can act as inhibitors of crystal growth by interacting with the crystal lattice and blocking nucleation sites^36^. Previous studies have shown that the disordered proteins such as N16 may offer conformational freedom and structural adaptation that allowed protein–protein or protein–mineral interactions^37^. It is noteworthy that although ESM-P and ESM-N possess similar secondary structures, they are likely composed of different sets of proteins. It is reported that proteins from prismatic and nacreous layers of *Pinctada margaritifera*, a close species with *P.fucata*, have very different protein repertoires^38^. It should also be noted that the CD spectra of proteins in solution are not necessary representative of the molecule conformation once entrapped in crystals. Thus, we infer that ESM-P and ESM-N have different effects on the crystallization process, resulting in different distribution patterns. From the point view of crystal growth, at low concentration of proteins, CaCO_3_ crystal growth in this case may follow classical crystallization pathway, i.e., ion-by-ion pathway instead of nanoparticle assembly pathway occurring in natural nacre^39^. Based on the pH-drop assay, ESM-P proteins have stronger inhibitory effects on the initial CaCO_3_ precipitation than ESM-N. ESM-P is protein fraction extracted from the calcite prismatic layers and the roles of these proteins are related to regulate calcite growth *in vivo*. By comparison, ESM-N is protein fraction extracted from the aragonite nacreous layers and the roles of these proteins are related to aragonite growth *in vivo*. Therefore, in the *in vitro* calcite growth assay, it is reasonable that ESM-P has more control over calcite growth than ESM-N. However, how specifically these proteins interact with minerals, which specific stages they take action, and what is the relationship between protein distribution and crystal formation are still yet to be addressed.

It is intriguing that how the heterogeneous distribution of proteins affect the mechanical properties of biominerals. Previous studies have demonstrated that cobble-like polygonal nanograins are basic building blocks to construct aragonite platelets into the nacre of red abalone. In addition, the deformability of the aragonite platelets together with the crack deflection, aragonite platelet slip, and organic adhesive interlayer contribute to the nacre’s fracture toughness^40^. In contrast, the outer prismatic layer does not show these crack diversion mechanisms. In the shell of a scallop, microcracks formed in the middle and bottom layers of the shell from microindentations show crack patterns forming along lamellar interfaces, which reduce the crack length and diffuse crack energy in three dimensions^41^. The aragonite nanoparticles are readily oriented and assembled into pseudo single-crystal aragonite platelets *via* screw dislocation and amorphous aggregation, which are two dominant mediating mechanisms between nanoparticles during biomineralization^42^. Moreover, Xiaodong Li *et al.* reveal that the unique nanoparticle−biopolymer arrangement renders the third-order lamellae a unique plasticity that reduces stress concentration and dissipates energy spatially in the conch shell’s crossed-lamellar structure. The biopolymer between nanoparticles serves as a lubricant to mediate the particle rotation, managing stress at the nanoscale^43^. Our results can explain these findings in some ways, namely, heterogeneous distribution of proteins inside crystals may occur in natural biominerals, rendering these biominerals heterogeneous mechanic properties and novel energy dissipation mechanisms. The interfaces between inorganic parts and organic parts may contribute significantly to these properties and mechanisms.

Together, the method provides a direct approach to observe the protein distribution in crystals without significantly altering the crystal formation process. Although the resolution under a conventional optical microscope is limited compared to electron microscopy, using high-resolution imaging techniques such as STORM^44^ may increase the resolution to observe the protein self-assembly process in crystals. Furthermore, real time observation of crystallization process in the presence of dye-labelled proteins may also provide more information about protein-mineral interactions during biomineralization process.

## Conclusions

In summary, by using FITC-labeled proteins extracted from the shell of *Pinctada fucata* as the additives, a heterogeneous distribution of biomineral-associated matrix proteins inside synthetic calcite at the microscale was observed for the first time according to the fluorescence intensity. The distribution may be related to their secondary structures and functions. This study shows the use of small-molecule dye-labeled matrix proteins as the additives for the visualization of protein distributions in crystals. Moreover, it indicates that different distribution patterns of proteins may occur in natural biominerals and may inspire synthesis of biocomposite materials.

## Additional Information

**How to cite this article**: Liu, C. *et al.* Heterogeneous distribution of dye-labelled biomineralizaiton proteins in calcite crystals. *Sci. Rep.* **5**, 18338; doi: 10.1038/srep18338 (2015).

## Supplementary Material

Supplementary Information
