# Supplementary material for: Heterogeneous distribution of dye-labelled biomineralizaiton proteins in calcite crystals
Source: Sci Rep. 2015 Dec 17;5:18338. doi: 10.1038/srep18338 (PMC4682127; doi:10.1038/srep18338)
Supplement: Supplementary Information [file srep18338-s1.pdf]

Supporting Information

Heterogeneous distribution of dye-labelled biomineralizaiton proteins in calcite crystals

Chuang Liu[1,2], *Liping Xie*[1], *Rongqing Zhang*[1] *

[1]Institute of Marine Biotechnology, Collaborative Innovation Center of Deep Sea Biology, School of Life Sciences, Tsinghua University, Beijing 100084 China,

E-mail: rqzhanglab@mail.tsinghua.edu.cn

[2]Tsinghua-Peking Joint Center for Life Sciences, School of Life Sciences, Tsinghua University, Beijing 100084 China

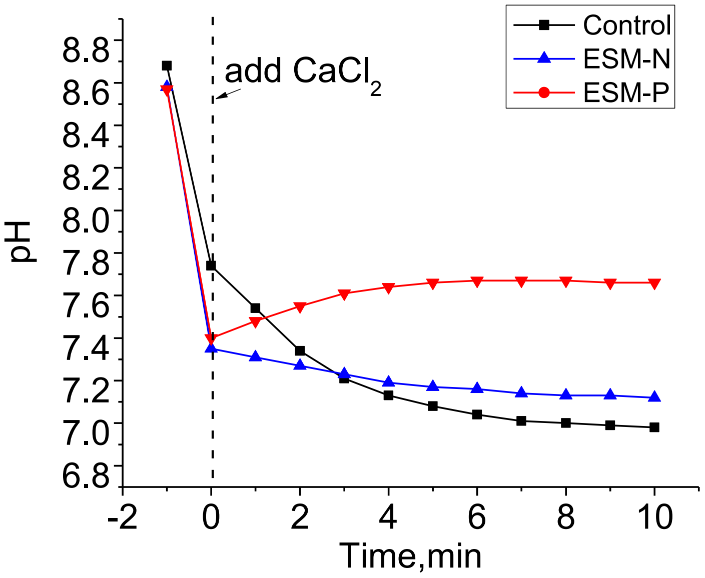

Figure S1. Inhibition of calcium carbonate precipitation assay The protein solution (200 μL, ~25 μg•mL^{-1}) was mixed with 10 mL of 40 mM $NaHCO_3$, pH 8.5. After the addition of 10 mL of 40 mM $CaCl_2$ to the mixture, the reaction was

monitored by recording the pH changes in the solutions per minute for 10 min. For the control, the protein solution was replaced with 200 μL DI water.

Table S1. The XRD parameter of different samples

Sample	FWHM (°)	Diffraction angle (2θ)
Control	0.194	29.330
ESM-P	0.215	29.424
ESM-N	0.198	29.325

Note: FWHM= full width at half maximum
